# Short‐ and Long‐Term Outcomes of Single‐Incision Laparoscopic Hepatectomy: A Matched Cohort Analysis

**DOI:** 10.1111/ases.70271

**Published:** 2026-03-12

**Authors:** Shu Aoyama, Takehiro Noda, Hirofumi Akita, Masahiro Umezu, Daisuke Taguchi, Yosuke Mukai, Kazuki Sasaki, Shinichiro Hasegawa, Daisaku Yamada, Yoshito Tomimaru, Tadafumi Asaoka, Hidenori Takahashi, Shogo Kobayashi, Junzo Shimizu, Yuichiro Doki, Hidetoshi Eguchi

**Affiliations:** ^1^ Department of Gastroenterological Surgery, Graduate School of Medicine The University of Osaka Suita Japan; ^2^ Department of Gastroenterological Surgery Osaka Keisatsu Hospital Osaka Japan; ^3^ Department of Gastroenterological Surgery Osaka International Cancer Institute Osaka Japan; ^4^ Department of Gastroenterological Surgery Toyonaka Municipal Hospital Toyonaka Japan

**Keywords:** hepatectomy, hepatocellular carcinoma, single‐incision laparoscopic surgery

## Abstract

**Introduction:**

Single‐incision laparoscopic hepatectomy has been introduced to reduce surgical invasiveness, but its short‐term and long‐term outcomes in malignant cases have not been fully elucidated. This study aimed to evaluate its impact on short‐ and long‐term outcomes of laparoscopic hepatectomy.

**Methods:**

This retrospective study included patients who underwent laparoscopic partial hepatectomy or left lateral sectionectomy for tumors with diameters < 5 cm, located in segments 2–6, between July 2009 and April 2019. Single‐incision and multi‐port procedures were matched one‐to‐one based on clinical factors, and short‐term outcomes were compared. Long‐term outcomes were also analyzed for hepatocellular carcinoma cases.

**Results:**

A total of 136 patients were included: 20 in the single‐incision group (17 with hepatocellular carcinoma) and 116 in the multi‐port group (69 with hepatocellular carcinoma). Within the matched cohort, compared to the multi‐port group, the single‐incision group showed comparable operative time (179 min in the single‐incision group vs. 185 min in the multi‐port group, *p* = 0.133) and blood loss (9 vs. 20 mL, *p* = 0.076). These groups did not significantly differ in postoperative hospital stay (12 vs. 12 days, *p* > 0.99) or complication rate (5.0% vs. 10.0%, *p* > 0.99). Among matched hepatocellular carcinoma cases, the two groups showed comparable 5‐year disease‐free survival (52.9% vs. 40.5%, *p* = 0.653) and overall survival (82.4% vs. 68.2%, *p* = 0.415).

**Conclusions:**

Compared with the multi‐port approach, single‐incision laparoscopic hepatectomy showed comparable and equivalent short‐ and long‐term outcomes. This procedure can be considered a safe and effective option for selected cases.

## 1 Introduction

Single‐incision laparoscopic surgery (SILS) was developed to reduce postoperative pain and wound‐related complications in laparoscopic surgery. While initially applied to simple surgeries, such as appendectomy [1], its application has expanded over time to a wide range of procedures, including cholecystectomy and nephrectomy [2]. This procedure has now been adopted for both benign conditions and malignant diseases. In addition to offering cosmetic advantages, SILS has yielded favorable long‐term outcomes—with SILS‐treated cases showing prognoses comparable to those following conventional multi‐port laparoscopic surgery in some malignant diseases, including colorectal and gastric cancer [3, 4, 5].

Several reports have suggested that single‐incision laparoscopic hepatectomy (SILH) leads to short‐term surgical outcomes that are comparable to those following multi‐port laparoscopic hepatectomy (MPLH) [6, 7]. However, most of these reports were retrospective and included a small number of cases, with no or limited adjustment for patient background; thus, the findings may have been influenced by differences in baseline characteristics and potential bias in treatment selection. It remains to be clarified whether SILH and MPLH yield comparable short‐term surgical outcomes under well‐balanced patient backgrounds.

Hepatocellular carcinoma (HCC) is the most common type of primary liver cancer, and hepatic resection is the first‐line treatment for resectable HCC [8, 9]. In recent years, MPLH has been widely adopted for HCC [10, 11, 12]. Compared to conventional open surgery for HCC, MPLH is reportedly associated with reduced intraoperative blood loss and shorter postoperative hospital stays, while yielding comparable long‐term outcomes in terms of disease‐free survival (DFS) and overall survival (OS) [13, 14]. On the other hand, only scarce data are available regarding the long‐term outcomes of SILH for HCC [15, 16]. Notably, the few studies of SILH for HCC have included limited indications, and have performed comparisons with MPLH without adjustment for baseline characteristics. Therefore, it remains uncertain whether SILH and MPLH have comparable long‐term outcomes.

We performed the first SILH procedure in Japan, and have since accumulated a series of such cases [17, 18]. In this study, we aimed to evaluate the impact of SILH on surgical outcomes for laparoscopic liver resection, and to investigate its association with long‐term outcomes in patients with HCC. We performed propensity score matching (PSM) to adjust for differences in patient background and channeling bias.

## 2 Materials and Methods

### 2.1 Study Design and Population

This study is a single‐center, retrospective observational study. The indications for SILH at our institution are non‐anatomical partial hepatectomy in the left lobe (S2/3/4), right anteroinferior segment (S5), or right posteroinferior segment (S6) where mobilization of the right lobe is not required, and left lateral sectionectomy, for tumors < 5 cm in diameter. Based on this practice, in the present study, we included patients who underwent laparoscopic hepatectomy (either partial hepatectomy or left lateral sectionectomy) for S2–6, at The University of Osaka Hospital, between July 2009 and April 2019. These patients were divided into two groups based on whether they underwent SILH or MPLH, and we compared patients' characteristics and surgical outcomes between these groups. Cases involving concurrent operations on other organs, except gallbladder, were excluded. Patient data were retrospectively collected from medical records. HCC cases were staged based on the sixth edition of the General Rules for the Clinical and Pathological Study of Primary Liver Cancer [19]. Liver cirrhosis was diagnosed via histological examination of the non‐tumorous liver tissue from the resected specimen. Postoperative complications were graded using the Clavien–Dindo classification (Version 2.0) [20]. For the HCC cases, we also analyzed OS and DFS following hepatectomy.

### 2.2 Surgical Procedure and Patient Follow‐Up

Details of the SILH procedure are described in our previous report [17]. Briefly, the patient was placed in the reverse Trendelenburg position, a 2.5‐cm skin incision was made at the umbilicus, and one 12‐mm trocar and two 5‐mm trocars were inserted through that incision using an access device. In some cases, an additional 5‐mm port was created. Intraabdominal pressure was maintained at 8 mmHg using an automatic CO_2_ insufflator. We performed 10‐mm laparoscopic ultrasonography to identify lesions and confirm the absence of other tumors in the liver. Next, the operation was carefully performed, without directly touching the tumor, and with sufficient margins to ensure complete removal. Ultrasonography was used to confirm the tumor's cut margin and the relationship to major vessels at the cutline. Liver resection was performed using a harmonic scalpel (Ethicon), and a saline‐linked sealing dissector was used to seal the major vessels. Subsequently, the resected specimen was extracted through the umbilical incision with a pouch. Patients were discharged after all drains had been removed, and their general condition had stabilized.

After hepatectomy for HCC, patients underwent follow‐up examinations every 3–6 months in the outpatient clinic. These visits included measurement of tumor markers, including alpha‐fetoprotein and des‐gamma‐carboxy prothrombin, and surveillance using abdominal ultrasound or abdominal computed tomography. The attending physician adjusted the follow‐up intervals and the types of examinations, as needed, based on the patient's general condition and risk of recurrence. When recurrence was suspected on surveillance imaging, magnetic resonance imaging was performed to establish a comprehensive diagnosis. The median follow‐up period for HCC cases in this study was 75.0 months.

### 2.3 Statistical Analysis

Descriptive statistics were presented as mean ± standard deviation, or median with interquartile range. Associations between the two groups were evaluated using Fisher's exact test for categorical variables, and the Mann–Whitney *U* test or *t*‐test for continuous variables. OS and DFS were analyzed using the Kaplan–Meier method, and differences between survival curves were assessed using the log‐rank test. A two‐sided *p*‐value of < 0.05 was considered statistically significant. No data were missing for the variables included in the analysis. To adjust for confounders, PSM was conducted, using propensity scores calculated using a logistic regression model. For analysis of surgical outcomes, the covariates included the presence of liver cirrhosis, diagnosed liver tumor, tumor size (> 2 cm), and tumor number (solitary or multiple). For analysis of the prognosis of HCC cases, we included the presence of liver cirrhosis, tumor size (> 2 cm), tumor number (solitary or multiple), and the presence of microscopic vascular invasion. These covariates were selected based on variables considered to influence the surgical selection of SILH and prognostic factors for resected HCC described in previous reports [ 21], taking into account their clinical relevance. *Optimal matching* without replacement was performed at a 1:1 ratio without applying a caliper. The standardized mean difference (SMD) was calculated to assess balance, with an SMD < 0.1 indicating adequate balance. All statistical analyses were performed using R (version 4.3.2; The R Project for Statistical Computing), and PSM was conducted using the *matchit* function in the *MatchIt* package in R.

### 2.4 Ethics Statement

This study was approved by the Human Ethics Review Committee of The University of Osaka Hospital (Hospital certificate number: 08226), and was conducted in accordance with the 1964 Helsinki Declaration and its later amendments. Written informed consent was obtained from all the participants.

## 3 Results

This study comprised a total of 136 cases, including 116 MPLH cases and 20 SILH cases. In six (30%) of the SILH cases, an additional port was added. Table 1 summarized the patients' characteristics and surgical outcomes for each group. The MPLH and SILH groups did not significantly differ in age, sex, body mass index, or American Society of Anesthesiologists' physical status. These groups also did not significantly differ in the distribution of diagnosed diseases—the MPLH group included primary liver cancer (81 cases, 69.8%), metastatic liver tumor (27 cases, 23.3%), and other diseases (8 cases, 6.9%); while the SILH group included primary liver cancer (17 cases, 85.0%), metastatic liver tumor (1 case, 5.0%), and other disease (2 cases, 10.0%). The proportion of patients with chronic hepatitis C tended to be higher in the SILH group, whereas the proportion of patients with chronic hepatitis B was comparable between the groups. Compared to the MPLH group, the SILH group included a significantly higher proportion of patients with liver dysfunction classified as Child‐Pugh class B (2.6% in the MPLH group vs. 15.0% in the SILH group, *p* = 0.041), and a significantly greater proportion of patients with cirrhosis (10.3% vs. 45.0%, *p* < 0.001). The groups had similar proportions of patients with multiple tumors (14 cases, 12.1% vs. 3 cases, 15.0%, *p* = 0.717) and the proportions of multiple resections were also similar between the two groups (9.5% vs. 10.0%, *p* ≥ 0.99). In both groups, cases involving multiple resections showed longer operative times than single resections (median operative time: 205 min for single resection vs. 294 min for multiple resections in the MPLH group, and 164 min vs. 194 min in the SILH group). The operative methods of multiple resections in the SILH group were S2 and S3 partial resection and S2 and S4 partial resection. The maximum tumor size tended to be slightly smaller in the SILH group compared to the MPLH group (20 mm vs. 19 mm, *p* = 0.065). The MPLH group exhibited a significantly higher rate of intraoperative total hepatic inflow occlusion using the Pringle maneuver (54.3% vs. 10.0%, *p* ≤ 0.001). Comparison of surgical outcomes between the two groups revealed that compared to the MPLH group, the SILH group had a significantly shorter operation time (218 min vs. 179 min, *p* = 0.003) and less intraoperative blood loss (50 mL vs. 9 mL, *p* = 0.004). The two groups did not significantly differ in the rate of conversion to laparotomy (6.0% vs. 10.0%, *p* = 0.620), the number of analgesic administrations within the first 24 h postoperatively (1 time vs. 1 time, *p* = 0.416), postoperative hospital stay (12 days vs. 12 days, *p* = 0.505), or the incidence of postoperative complications (16.4% vs. 5.0%, *p* = 0.306). Complications of Clavien–Dindo grade III or higher were not observed in the SILH group, but occurred in two cases (1.7%) in the MPLH group—including one case of atrial fibrillation requiring electrical cardioversion, and one case of bile leakage. No surgery‐related mortality occurred in either group.

**TABLE 1:** Patient characteristics and surgical outcomes of all cases.

		MPLH, *n* (%)	SILH, *n* (%)	*p*
		*n* = 116	*n* = 20	
Age, years	Median (IQR)	70 (63–77)	70 (65–77)	0.733
Sex				
Male		75 (64.7)	14 (70.0)	0.801
Female		41 (35.3)	6 (30.0)	
Body mass index	Mean ± SD	22.8 ± 3.84	22.4 ± 2.74	0.708
ASA‐PS				
1		20 (17.2)	2 (10.0)	0.236
2		83 (71.6)	13 (65.0)	
3		13 (11.2)	5 (25.0)	
Disease				
Primary liver tumor		81 (69.8)	17 (85.0)	0.127
Metastatic liver tumor		27 (23.3)	1 (5.0)	
Others		8 (6.9)	2 (10.0)	
Hepatitis				
HBs Ag		30 (25.9)	6 (30.0)	0.785
HCV Ab		37 (31.9)	11 (55.0)	0.074
Child‐Pugh classification				
A		113 (97.4)	17 (85.0)	0.041
B		3 (2.6)	3 (15.0)	
Liver cirrhosis		12 (10.3)	9 (45.0)	< 0.001
Tumor number				
Single		102 (87.9)	17 (85.0)	0.717
Multiple		14 (12.1)	3 (15.0)	
Number of resections				
1		105 (90.5)	18 (90.0)	> 0.99
≥ 2		11 (9.5)	2 (10.0)	
Maximum tumor size, mm	Median (IQR)	20 (17–32)	19 (12–24)	0.065
Type of liver resection				
Lateral sectionectomy		17 (14.7)	1 (5.0)	0.472
Partial resection		99 (85.3)	19 (95.0)	
Pringle maneuver		63 (54.3)	2 (10.0)	< 0.001
History of abdominal surgery		60 (51.7)	9 (45.0)	0.634
History of liver resection		21 (18.1)	4 (20.0)	0.763
Operation time, min	Median (IQR)	218 (172–288)	179 (120–200)	0.003
Blood loss, mL	Median (IQR)	50 (10–150)	9 (5–35)	0.004
Conversion to laparotomy		7 (6.0)	2 (10.0)	0.620
Analgesic use within 24 h postoperatively		1 (1–3)	1 (0–3)	0.416
Postoperative hospital stay, days	Median (IQR)	12 (10–14)	12 (9–13)	0.505
Postoperative complications				
All		19 (16.4)	1 (5.0)	0.306
Clavien‐Dindo I or II		17 (14.7)	1 (5.0)	
Clavien‐Dindo≥ III		2 (1.7)	0	
Mortality		0	0	—

Abbreviations: ASA‐PS, American Society of Anesthesiologists' physical status; HBs Ag, hepatitis B surface antigen; HCV Ab, hepatitis C virus antibody; IQR, interquartile range; MPLH, multi‐port laparoscopic hepatectomy; SD, standard deviation; SILH, single‐incision laparoscopic hepatectomy.

We conducted 1:1 PSM using the SILH group and matched controls from the MPLH group. Table 2 summarized the patients' backgrounds after matching. The groups differed in Child–Pugh classification, presence of liver cirrhosis, and tumor size before matching, and these factors became comparable between groups after matching. Although the usage rate of the Pringle maneuver remained higher in the MPLH group, this difference was not statistically significant. Other baseline characteristics were also balanced between the two groups. Regarding surgical outcomes in the matched cohort, the MPLH and SILH groups did not significantly differ in operation time (185 min vs. 179 min, *p* = 0.133) or intraoperative blood loss (20 mL vs. 9 mL, *p* = 0.076). As observed in the pre‐matching cohort, in the matched cohort, the two groups were comparable in terms of postoperative analgesics use, postoperative hospital stay and the incidence of postoperative complications.

**TABLE 2:** Patient characteristics and surgical outcomes of the matched cases.

		MPLH, *n* (%)	SILH, *n* (%)	*p*
		*n* = 20	*n* = 20	
Age in years	Median (IQR)	71 (63–81)	70 (65–77)	> 0.99
Sex				
Male		10 (50.0)	14 (70.0)	0.333
Female		10 (50.0)	6 (30.0)	
Body mass index	Mean ± SD	22.6 ± 4.04	22.4 ± 2.74	0.699
ASA‐PS				
1		2 (10.0)	2 (10.0)	> 0.99
2		14 (70.0)	13 (65.0)	
3		4 (20.0)	5 (25.0)	
Disease				
Primary liver tumor		17 (85.0)	17 (85.0)	> 0.99
Metastatic liver tumor		1 (5.0)	1 (5.0)	
Others		2 (10.0)	2 (10.0)	
Hepatitis				
HBs Ag		4 (20.0)	6 (30.0)	0.716
HCV Ab		8 (40.0)	11 (55.0)	0.527
Child‐Pugh classification				
A		20 (100)	17 (85.0)	0.231
B		0	3 (15.0)	
Liver cirrhosis		9 (45.0)	9 (45.0)	> 0.99
Tumor number				
Single		16 (80.0)	17 (85.0)	> 0.99
Multiple		4 (20.0)	3 (15.0)	
Number of resections				
1		16 (80.0)	18 (90.0)	0.661
≥ 2		4 (20.0)	2 (10.0)	
Maximum tumor size, mm	Median (IQR)	19 (17–27)	19 (12–24)	0.243
Type of liver resection				
Lateral sectionectomy		2 (10.0)	1 (5.0)	> 0.99
Partial resection		18 (90.0)	19 (95.0)	
Pringle maneuver		8 (40.0)	2 (10.0)	0.065
History of abdominal surgery		7 (35.0)	9 (45.0)	0.748
History of liver resection		4 (20.0)	4 (20.0)	> 0.99
Operation time, min	Median (IQR)	185 (164–250)	179 (120–200)	0.133
Blood loss, mL	Median (IQR)	20 (10–54)	9 (5–35)	0.076
Conversion to laparotomy		1 (5.0)	2 (10.0)	> 0.99
Analgesic use within 24 h postoperatively		2 (0–3)	1 (0–3)	0.496
Postoperative hospital stay, days	Median (IQR)	12 (9–14)	12 (9–13)	> 0.99
Postoperative complications				
All		2 (10.0)	1 (5.0)	> 0.99
Clavien‐Dindo I or II		2 (10.0)	1 (5.0)	
Clavien‐Dindo≥ III		0	0	
Mortality		0	0	—

Abbreviations: ASA‐PS, American Society of Anesthesiologists' physical status; HBs Ag, hepatitis B surface antigen; HCV Ab, hepatitis C virus antibody; IQR, interquartile range; MPLH, multi‐port laparoscopic hepatectomy; SD, standard deviation; SILH, single‐incision laparoscopic hepatectomy.

**FIGURE 1:**
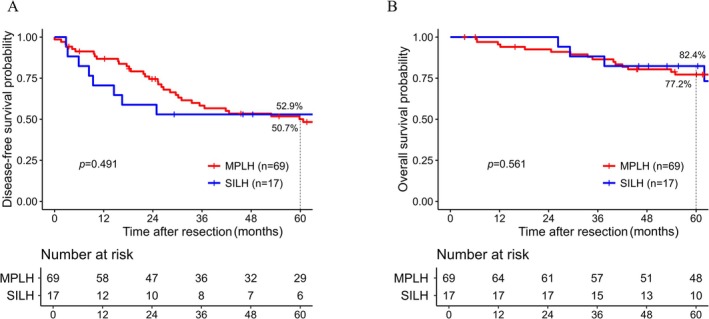
Prognosis following laparoscopic hepatectomy among all hepatocellular carcinoma cases. (A) Disease‐free survival. (B) Overall survival. Red lines represent MPLH cases. Blue lines represent SILH cases. MPLH, multi‐port laparoscopic hepatectomy; SILH, single‐incision laparoscopic hepatectomy.

This study comprised a total of 86 HCC cases, including 69 in the MPLH group and 17 in the SILH group. Table 3 summarized the patients' backgrounds for HCC cases. The groups did not significantly differ in age, sex, or underlying hepatitis. The SILH group tended to have a higher proportion of Child‐Pugh class B cases (2.9% vs. 17.6%, *p* = 0.051) and included a significantly higher proportion of patients with cirrhosis (17.4% vs. 47.1%, *p* = 0.021). Additionally, tumor size was significantly smaller in the SILH group compared to the MPLH group (22 mm vs. 18 mm, *p* = 0.022). Other clinicopathological factors did not significantly differ between the two groups, including the rate of positive surgical margins. Comparison of prognosis between the two groups revealed no significant difference in 5‐year DFS (50.7% in the MPLH group vs. 52.9% in the SILH group, *p* = 0.491) (Figure 1A), or 5‐year OS (77.2% vs. 82.4%, *p* = 0.561) (Figure 1B).

**TABLE 3:** Baseline characteristics of all the hepatocellular carcinoma cases.

		MPLH, *n* (%)	SILH, *n* (%)	*p*
		*n* = 69	*n* = 17	
Age, years	Median (IQR)	71 (63–79)	75 (66–78)	0.965
Sex				
Male		48 (69.6)	11 (64.7)	0.733
Female		21 (30.4)	6 (35.3)	
Hepatitis				
HBs Ag		22 (31.9)	5 (29.4)	> 0.99
HCV Ab		34 (49.3)	11 (64.7)	0.290
Child‐Pugh classification				
A		67 (97.1)	14 (82.4)	0.051
B		2 (2.9)	3 (17.6)	
Liver cirrhosis		12 (17.4)	8 (47.1)	0.021
Alpha‐fetoprotein, ng/mL	Median (IQR)	6 (3–23)	5 (3–48)	0.659
Des‐gamma‐carboxy Prothrombin, mAU/mL	Median (IQR)	56 (28–333)	35 (16–180)	0.488
Tumor number				
Single		62 (89.9)	14 (82.4)	0.407
Multiple		7 (10.1)	3 (17.6)	
Maximum tumor size, mm	Median (IQR)	22 (17–32)	18 (12–23)	0.022
History of liver resection for HCC		16 (23.2)	4 (23.5)	> 0.99
Edmondson classification				
1		9 (13.0)	3 (17.6)	0.342
2		40 (58.0)	9 (52.9)	
3		17 (24.6)	3 (17.6)	
4		2 (2.9)	0	
Others		1 (1.4)	2 (11.8)	
Microscopic vascular invasion		13 (18.8)	2 (11.8)	0.725
Microscopic intrahepatic metastasis		7 (10.1)	0	0.336
Positive surgical margin		2 (2.9)	0	> 0.99

Abbreviations: HBs Ag, hepatitis B surface antigen; HCV Ab, hepatitis C virus antibody; IQR, interquartile range; MPLH, multi‐port laparoscopic hepatectomy; SILH, single‐incision laparoscopic hepatectomy.

**FIGURE 2:**
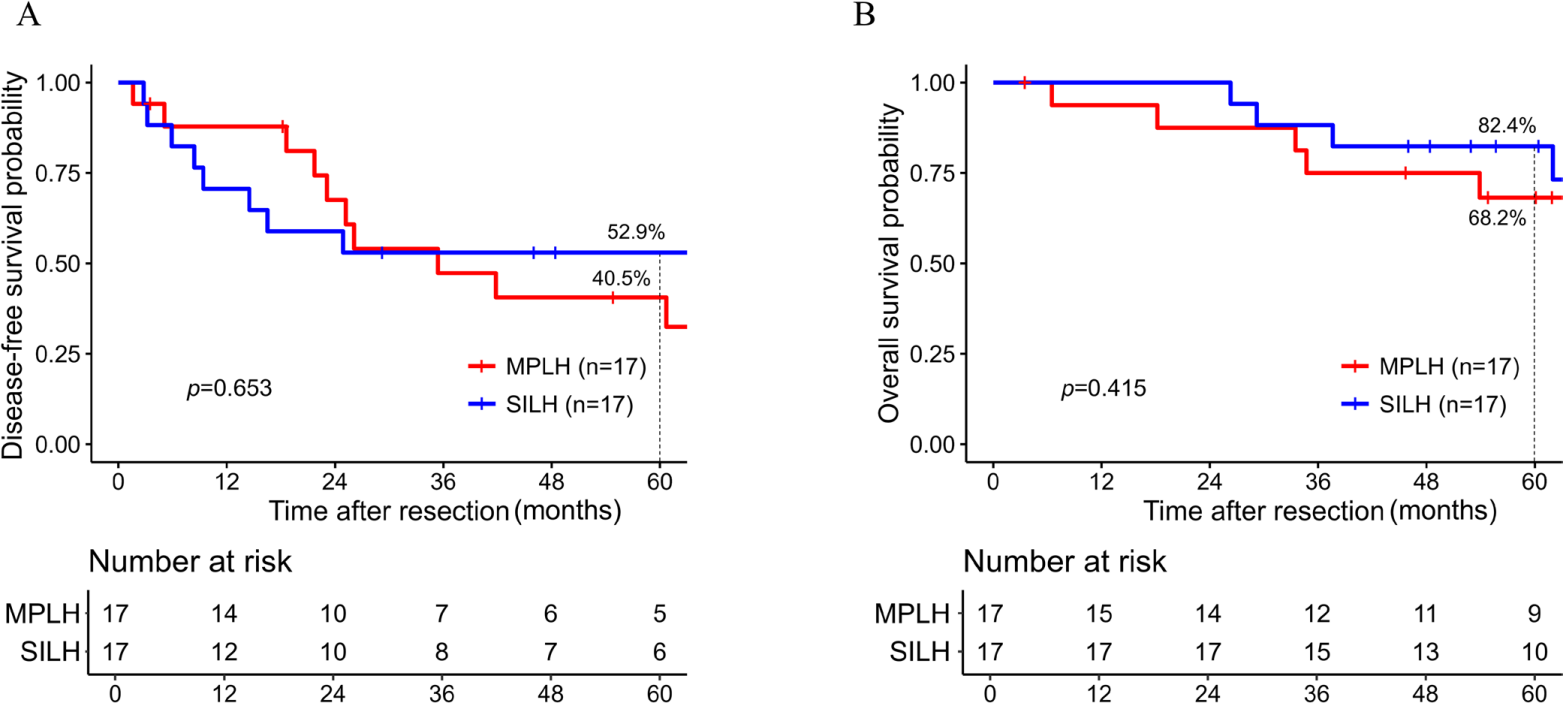
Prognosis following laparoscopic hepatectomy among the matched hepatocellular carcinoma cases. (A) Disease‐free survival. (B) Overall survival. Red lines represent MPLH cases. Blue lines represent SILH cases. MPLH, multi‐port laparoscopic hepatectomy; SILH, single‐incision laparoscopic hepatectomy.

Table 4 summarized the patients' characteristics after 1:1 PSM for HCC cases. The groups differed in Child–Pugh classification, presence of liver cirrhosis, and tumor size before matching, and these factors became comparable between groups in the matched cohort. Other clinicopathological baseline factors were comparable between the groups. As in the pre‐matching cohort, in the matched cohort, the two groups did not significantly differ in 5‐year DFS (40.5% vs. 52.9%, *p* = 0.653) or 5‐year OS (68.2% vs. 82.4%, *p* = 0.415) (Figure 2).

**TABLE 4:** Baseline characteristics of the matched hepatocellular carcinoma cases.

		MPLH, *n* (%)	SILH, *n* (%)	*p*
		*n* = 17	*n* = 17	
Age, years	Median (IQR)	75 (70–82)	75 (66–78)	0.343
Sex				
Male		8 (47.1)	11 (64.7)	0.491
Female		9 (52.9)	6 (35.3)	
Hepatitis				
HBs Ag		4 (23.5)	5 (29.4)	> 0.99
HCV Ab		12 (70.6)	11 (64.7)	> 0.99
Child‐Pugh classification				
A		16 (94.1)	14 (82.4)	0.601
B		1 (5.9)	3 (17.6)	
Liver cirrhosis		8 (47.1)	8 (47.1)	> 0.99
Alpha‐fetoprotein, ng/mL	Median (IQR)	6 (3–10)	5 (3–48)	0.358
Des‐gamma‐carboxy Prothrombin, mAU/mL	Median (IQR)	47 (28–104)	35 (16–180)	0.642
Tumor number				
Single		14 (82.4)	14 (82.4)	> 0.99
Multiple		3 (17.6)	3 (17.6)	
Maximum tumor size, mm	Median (IQR)	18 (17–26)	18 (12–23)	0.269
History of liver resection for HCC		4 (23.5)	4 (23.5)	> 0.99
Edmondson classification				
1		5 (29.4)	3 (17.6)	0.602
2		8 (47.1)	9 (52.9)	
3		4 (23.5)	3 (17.6)	
4		0	0	
Others		0	2 (11.8)	
Microscopic vascular invasion		1 (5.9)	2 (11.8)	> 0.99
Microscopic intrahepatic metastasis		2 (11.8)	0	0.485
Positive surgical margin		0	0	—

Abbreviations: HBs Ag, hepatitis B surface antigen; HCV Ab, hepatitis C virus antibody; IQR, interquartile range; MPLH, multi‐port laparoscopic hepatectomy; SILH, single‐incision laparoscopic hepatectomy.

## 4 Discussion

This study evaluated the surgical and long‐term outcomes of laparoscopic liver resection using SILH compared to MPLH. Within the matched cohort, SILH showed short‐term and long‐term outcomes that were comparable and equivalent to those of MPLH. These results indicate that SILH can be a feasible and oncologically acceptable approach, offering similar outcomes to MPLH even in malignant cases, such as HCC.

Several studies of malignancies other than HCC have reported comparable long‐term outcomes between patients treated with SILS versus conventional multi‐port laparoscopic surgery. For example, in a retrospective analysis of patients with colorectal cancer, Katsuno et al. compared 107 patients who underwent single‐incision laparoscopic colectomy with 107 matched patients who underwent multi‐port laparoscopic colectomy and reported comparable 5‐year OS between groups (100% with single‐port procedures vs. 95% with multi‐port procedures, *p* = 0.125) [4]. Similarly, several studies of gastric cancer have reported long‐term outcomes following SILS. Kang et al. analyzed early‐stage gastric cancer patients who underwent either single‐port or multi‐port laparoscopic distal gastrectomy [5]. With 378 patients in each group matched using PSM, they demonstrated comparable 5‐year OS between the two groups (95.8% with single‐port procedures vs. 94.2% with multi‐port procedures, *p* = 0.43). For primary liver cancers, there have been limited reports of the long‐term outcomes of SILH. Tsai et al. compared the prognosis of SILH (*n* = 22) and MPLH (*n* = 33) among HCC patients [15]. In their study, SILH was performed for tumors located in S5–6 or in the left lateral segment, and most procedures in the SILH group were either partial hepatectomy or left lateral sectionectomy. The median tumor size was 2.4 cm in S5–6. Within the SILH group, 22.2% of patients had liver cirrhosis. The two groups that underwent partial hepatectomy did not significantly differ in 5‐year OS (86.7% after MPLH vs. 69.2% after SILH, *p* = 0.819). Likewise, among patients who underwent left lateral sectionectomy for HCC, Wang et al. reported that SILH and MPLH were associated with comparable 1‐year DFS rates (73.9% vs. 70.7%, *p* = 0.82); however, their study had a relatively short follow‐up period [16]. The SILH group in their study did not include any patients with Child–Pugh class B and exhibited a median tumor size of 3.0 cm. Although these prior studies did not include statistical adjustment for baseline characteristics, the reported survival rates were comparable to those observed in our present study. In our cohort, DFS and OS did not significantly differ between the SILH group and the MPLH group either before and after adjustment using PSM. Moreover, the indications for SILH in our study did not substantially differ from those described in previous reports. Overall, the available findings suggest that when applied in appropriately selected patients, SILH can provide oncological outcomes comparable to those of MPLH. This means that surgeons can consider less invasive options without compromising long‐term efficacy.

The unadjusted analysis of short‐term outcomes in the present study revealed that the SILH group had a significantly shorter operative time and significantly less intraoperative blood loss, compared to the MPLH group. However, these differences were not statistically significant after PSM, suggesting possible channeling bias. Nevertheless, even after PSM, these outcomes were comparable between the SILH and MPLH groups. In both the unadjusted and matched analyses, the two groups were similar in terms of the incidence of postoperative complications and the length of postoperative hospital stay. Han et al. performed a retrospective unadjusted study involving 155 SILH cases and 91 MPLH cases, and reported a significantly shorter operative time in the SILH group (137 min vs. 231 min, *p* < 0.001), as well as significantly reduced intraoperative blood loss in the SILH group [22]. They found that the groups did not significantly differ in postoperative complication rates or length of hospital stay. The trends they observed regarding operative time and blood loss were consistent with the findings of our present unadjusted analysis, and may reflect differences in patient selection or background characteristics. Several systematic reviews and meta‐analyses have also suggested that SILH produces surgical outcomes that are comparable to or even better than those of MPLH [6, 23]; however, these analyses are based on unadjusted data from individual studies with relatively small sample sizes, and may be affected by channeling bias. Our present study demonstrated no significant differences in surgical outcomes between SILH and MPLH, even after adjustment for baseline characteristics using PSM, indicating that SILH may achieve surgical outcomes comparable to MPLH under appropriately selected conditions. These findings indicate that SILH can be a feasible and safe surgical option for minimally invasive liver resection in patients similar to those in our cohort, involving partial hepatectomy or left lateral sectionectomy for tumors < 5 cm located in segments 2–6. Of note, although limited in number, the SILH group included two cases involving multiple resections, comparable in proportion to the MPLH group. In both cases, the lesions were confined to the same lobe, suggesting that multiple resections may be safely performed in SILH when restricted to a single lobe. Additionally, recent reports have described the performance of SILS using new surgical platforms, such as the da Vinci SP system. Such platforms have already demonstrated favorable short‐term outcomes in some diseases, including colorectal and gastric cancer [24, 25]. These promising results suggest that SILH may become more widely adopted in the future.

The present study has several limitations. This was a single‐center retrospective analysis including a limited number of SILH cases. Therefore, the number of covariates included in the PSM model was restricted and some degree of residual confounding may have remained; however, the matched cohorts achieved an acceptable balance. Moreover, the indication for SILH was restricted to tumors in specific segments; therefore, the findings may not be applicable to tumors in other anatomical regions. Additionally, the use of an additional port in some SILH cases may have affected surgical outcomes. Finally, the small number of HCC cases included in the long‐term analysis may have limited the ability to detect differences in prognosis.

In conclusion, SILH and MPLH showed comparable and equivalent surgical and long‐term outcomes, including in patients with HCC. Even after adjustment using PSM, no significant differences were observed between groups. These findings suggest that SILH can be a feasible and effective alternative to MPLH in selected cases.

## Author Contributions

S.A. and T.N. contributed to the methodology and wrote the manuscript. H.A., M.U., D.T., Y.M., K.S., and S.H. contributed to the investigation and data curation. D.Y., Y.T., and T.A. supervised the clinical aspects of the study and provided critical feedback. H.T., S.K., and J.S. were involved in validation and interpretation of the data. Y.D. and H.E. conceptualized the study and supervised the entire project. All authors read and approved the final manuscript.

## Funding

The authors have nothing to report.

## Disclosure

The authors have nothing to report.

## Conflicts of Interest

The authors declare no conflicts of interest.

## Data Availability

The data that support the findings of this study are available from the corresponding author upon reasonable request.
